# Transcriptomics of a Giant Freshwater Prawn (*Macrobrachium rosenbergii*): *De Novo* Assembly, Annotation and Marker Discovery

**DOI:** 10.1371/journal.pone.0027938

**Published:** 2011-12-08

**Authors:** Hyungtaek Jung, Russell E. Lyons, Hung Dinh, David A. Hurwood, Sean McWilliam, Peter B. Mather

**Affiliations:** 1 Biogeosciences, Queensland University of Technology, Brisbane, Queensland, Australia; 2 Commonwealth Scientific and Industrial Research Organisation Livestock Industries, St Lucia, Queensland, Australia; Hospital for Sick Children, Canada

## Abstract

**Background:**

Giant freshwater prawn (*Macrobrachium rosenbergii* or GFP), is the most economically important freshwater crustacean species. However, as little is known about its genome, 454 pyrosequencing of cDNA was undertaken to characterise its transcriptome and identify genes important for growth.

**Methodology and Principal Findings:**

A collection of 787,731 sequence reads (244.37 Mb) obtained from 454 pyrosequencing analysis of cDNA prepared from muscle, ovary and testis tissues taken from 18 adult prawns was assembled into 123,534 expressed sequence tags (ESTs). Of these, 46% of the 8,411 contigs and 19% of 115,123 singletons possessed high similarity to sequences in the GenBank non-redundant database, with most significant (E value < 1e^–5^) contig (80%) and singleton (84%) matches occurring with crustacean and insect sequences. KEGG analysis of the contig open reading frames identified putative members of several biological pathways potentially important for growth. The top InterProScan domains detected included RNA recognition motifs, serine/threonine-protein kinase-like domains, actin-like families, and zinc finger domains. Transcripts derived from genes such as actin, myosin heavy and light chain, tropomyosin and troponin with fundamental roles in muscle development and construction were abundant. Amongst the contigs, 834 single nucleotide polymorphisms, 1198 indels and 658 simple sequence repeats motifs were also identified.

**Conclusions:**

The *M. rosenbergii* transcriptome data reported here should provide an invaluable resource for improving our understanding of this species' genome structure and biology. The data will also instruct future functional studies to manipulate or select for genes influencing growth that should find practical applications in aquaculture breeding programs.

## Introduction

Of the 200 or so aquaculture species, decapod crustaceans including prawns, lobsters and crabs contribute substantially to the US$60 billion global industry [1]. Amongst farmed crustaceans, the giant freshwater prawn (*Macrobrachium rosenbergii*) has increasingly become an aquaculture species of major commercial value, with revenue in Asia alone currently worth >US$1 billion annually [1]–[4]. Due to its high value, research is now focusing on improving the growth performance of farmed *M. rosenbergii* [2]–[6]. However, little is known about this species' basic biology and genome make-up so that they can be exploited to improve farm productivity of this species.

Genomics approaches are now being applied widely to elucidate genetic factors conferring economically significant traits and/or phenotypes and to manage genetic diversity in cultured crustacean species [7]–[10]. Whilst their application to cultured fish species has produced significant production gains, such gains are only beginning to be realized in penaeid species [11]–[15], and no detailed genetic analyses have yet been reported for *M. rosenbergii*. Such basic information is essential to better understand a species' biology and to devise strategies to improve productivity in culture. DNA microsatellites [16]–[18] and mitochondrial DNA sequence comparisons [19] have been used to examine the phylogeography of *M. rosenbergii* [20], [21] sampled from Asia and northern Australia and genes potentially associated with pathogen defence responses [22]–[24] and sexual maturation traits [25] have also been identified. However, more genome-wide or transcriptome-wide datasets have yet to be generated as a basis for functional genomics approaches [26]–[29] aimed at improving the aquaculture performance of this species.

Roche 454 Genome Sequencing FLX technology is particularly useful as a shotgun method for generating data broadly across novel genomes, and it is relatively cheap [26], [33], [31] and exceptionally accurate [26]–[31]. Here it was used to characterize the transcriptome of *M*. *rosenbergii* using cDNA prepared from mRNA isolated from muscle, ovary and testis tissues. Expressed sequence tag (EST) sequences generated were assembled and annotated with putative functions where possible, and database searches were performed to identify candidate protein domains, genes and gene families potentially involved with growth. A variety of markers potentially useful for genomic population studies including simple sequence repeats (SSRs) located within coding regions and single nucleotide polymorphisms (SNPs) detected amongst deep coverage sequence regions reads are also reported.

## Results and Discussion

### Roche 454 GS-FLX sequencing and contig assembly

**Figure 1:**
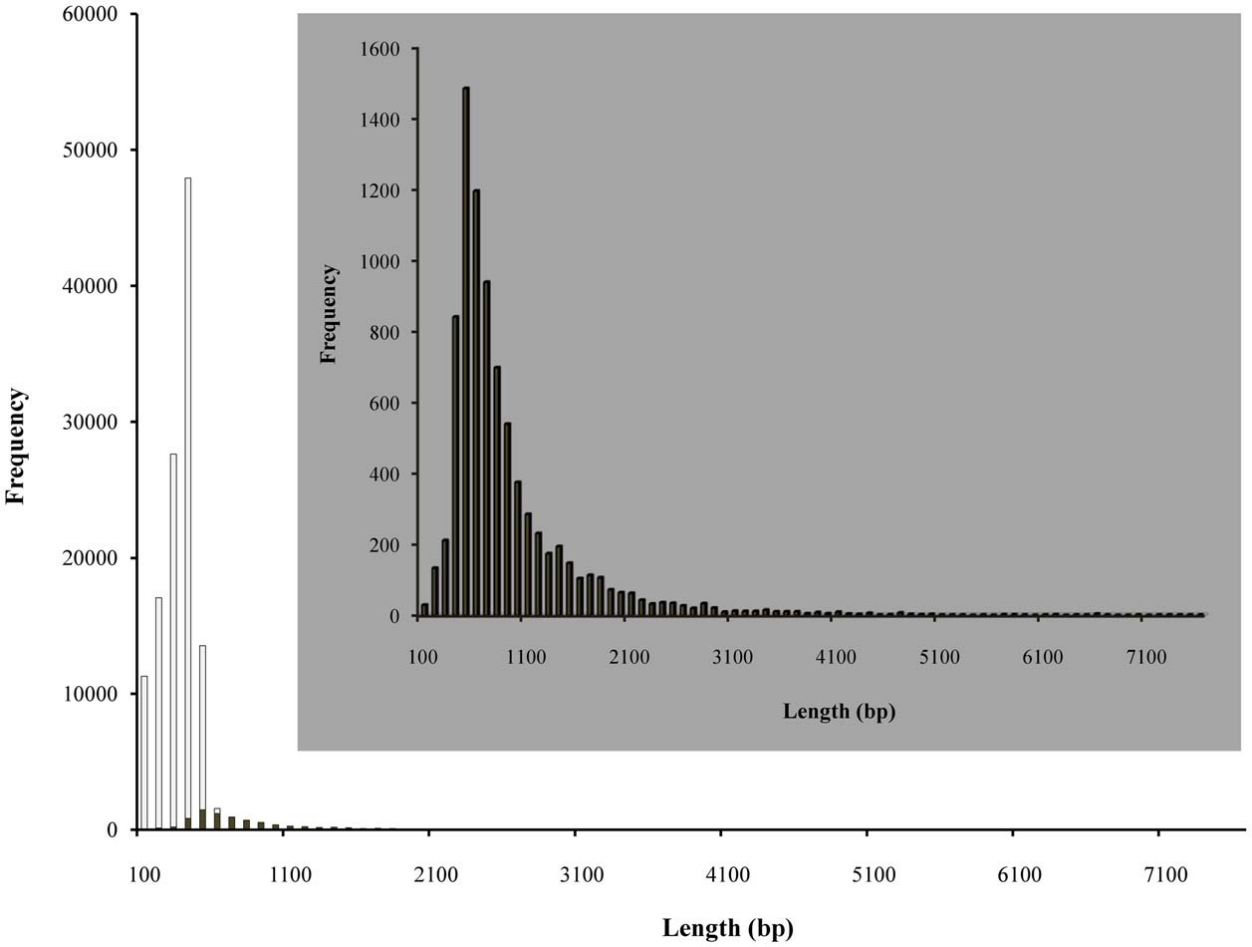
Summary of *M*. *rosenbergii* transcriptomic sequences. The contig sequences are represented by solid bars and the singleton sequences by open bars.

cDNA prepared to mRNA purified from muscle, ovary and testis tissues from *M. rosenbergii* were sequenced using the 454 GS-FLX platform. Sequences that passed basic quality standards were clustered and assembled *de novo*. In 454 sequencing run #1, a total of 121,214 EST sequences (total  =  36.45 Mb) were assembled from mRNA isolated from either muscle or ovary tissue sampled from 6 adult females and preserved in ethanol prior to analysis. Average EST length was 295 nucleotides (nt). Assembly of high quality ESTs generated 1983 contigs averaging 673 nt in length. Due to technical issues with the first 454 GS-FLX run, the expected amount of data (200 Mb) was not retrieved. Therefore a second 454 sequencing run was conducted to increase genomic data, including the addition of testis-derived RNA. In 454 sequencing run #2, a total of 666,517 EST sequences were assembled from mRNA isolated from muscle and ovary from 9 adult females and 3 adult male testis tissues and preserved in RNAlater solution (Ambion) prior to analysis. Eyestalk-derived RNA was also extracted, but ultimately excluded from sequencing run #2 as quality control indicators suggested it contained PCR and proteinase inhibitors leading to failure of cDNA fragmentation, as detected in the bioanalyzer traces (samples were not fragmented). For the remaining three tissue types, the average EST length was 311 nt in 454 sequencing run #2. After removing adaptor sequences, the combined run #1 and #2 dataset contained 244.37 Mb of sequence comprising 787,731 reads averaging 310 nt in length, and the average coverage depth was 29.85 sequences per nucleotide position (**Table 1**). This average EST read length is longer and the sequencing coverage depth is substantially higher than has been reported in similar 454 sequencing analyses in non-model species including Glanville fritillary (197 nt at 2.3 x coverage; [26]), flooded or rose gum (245 nt; [32]) or shore pine (306 nt at 3.6 x coverage; [33]). As shown in **Figure 1**, assembly of high quality *M. rosenbergii* EST sequences generated 8,411 contigs varying in length from 40 nt to 7,531 nt (average 845 nt; total 212,142,540 nt), with 5,724 (68%) being >500 nt in length. The long individual read lengths combined with the 29.85-fold average coverage contributed to this high proportion of long contig sequences. Singletons ranged from 50 nt to 773 nt in length (average 279 nt, total 32,228,442 nt) (**Figure 1**). To our knowledge, this is the first comprehensive study of the transcriptome of *M. rosenbergii*.

**Table 1:** Summary of 454 pyrosequencing, assembly and analysis of *M*. *rosenbergii* transcriptomic sequences.

Dataset name		All	Muscle	Ovary	Testis
Total number of bases (Mp)		244.37	114.38	86.06	43.94
Average read length (bp)		310	311	308	311
No. of reads	Total	787,731	367,379	279,393	140,959
	Assembled	645,837	323,044	189,771	112,271
	Singleton	115,123	33,622	77,455	24,995
	Repeat	276	136	197	77
No. of contigs	Total contigs	8,411	1,723	5,346	1004
	Average contigread length (bp)	845	1,027	796	848
	Largest contig (bp)	7,531	7,304	6,955	7,530
	No. of largeContigs > 500bp	5,724	1,171	3,559	683
Average coverage (x)		29.85	59.56	14.92	43.09

### Comparative analyses of ESTs

**Figure 2:**
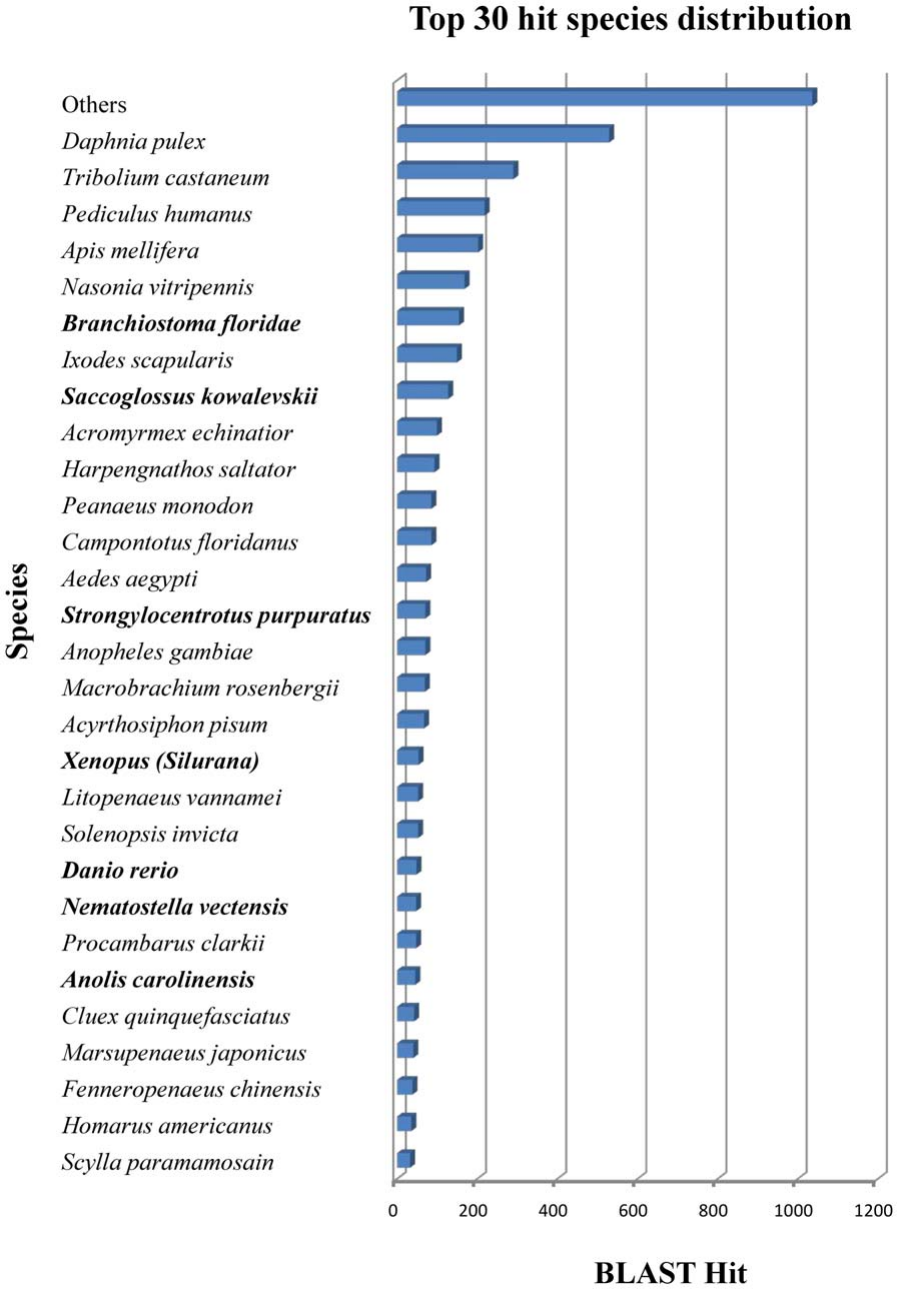
Top 30 hit species distribution based on BLASTx. E value cut-off is 1e^–5^ and top 30 hit species distribution of gene annotations showing high homology to the Arthropoda (Insecta and Crustacea) phylum with known genome sequences. Only contig sequences were used. Bold text indicates non-Arthropod homology.

From BLASTx searches of *M*. *rosenbergii* EST coding sequences, 3,757 of the 8,411 (46%) contigs and 21,965 of the 115,123 (19%) singletons possessed significant similarity (E value <1e^–5^) with proteins in the GenBank non-redundant (nr) database (**Table S1**). As might be expected, coding sequences in the majority of contigs (80%) and singletons (84%) matched well to crustacean and other arthropod proteins (**Figure 2**) which are in agreement with previous prawn studies [14], [15]. After redundant and ribosomal protein sequences were excluded, 2,448 contig and 10,627 singleton sequences were identified as putative genes based on BLASTx matches.

Species most represented in the BLASTx searches included some penaeid shrimps, crabs and freshwater and marine crayfish species including giant tiger shrimp (*Penaeus monodon*), green mud crab (*Scylla paramamosain*), fleshy shrimp (*Fenneropenaeus chinensis*), Kuruma shrimp (*Marsupenaeus japonicas*), white leg shrimp (*Litopenaeus vannamei*), red swamp crayfish (*Procambarus clarkia*), and American lobster (*Homarus americanus*). Similarities in EST coding sequences are indicative of close evolutionary relationship of *M. rosenbergii* with other crustaceans. Only a few contig (1.8%) or singleton (3.9%) coding sequences matched protein sequences reported for *M. rosenbergii*, and again this was expected due to the limited number of *M. rosenbergii* EST (2365) and protein sequences (373) currently available in the NCBI databases. The *M. rosenbergii* EST sequences generated here thus will vastly expand the number of genes identified in this species.

**Figure 3:**
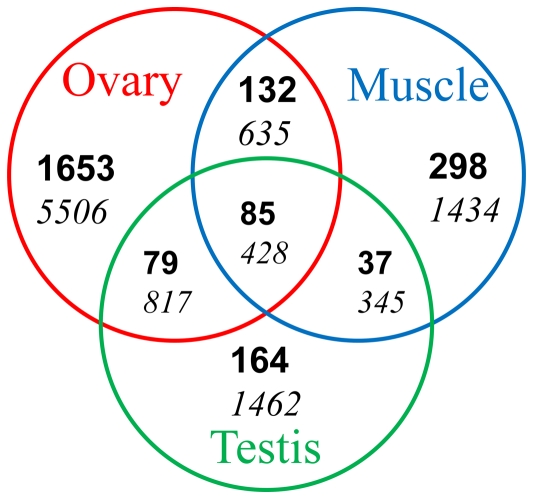
Comparative summary of *M*. *rosenbergii* transcriptomic sequences among three libraries. Putative sequence descriptions were counted using BLASTx results (E-value <1e^–5^) after excluding ribosomal proteins and redundant ones. Bold numbers indicate contigs and numbers in italics indicate singletons.

More putative gene ESTs were detected in mRNA isolated from ovary tissue than from muscle or testis tissue (**Figure 3**). Only around 4% of the 3,757 contigs and 14% of the 21,965 singletons significantly matched either predicted or hypothetical genes (E value <1e^–5^) due to the limited genomic information available for prawn species in the public database (**Table S1**). A significant number of *M*. *rosenbergii* ESTs did not possess coding sequences matching any sequences in the GenBank nr database which is not surprising for prawn EST studies [14], [15]. Whilst most of these likely represent ESTs spanning only untranslated mRNA regions, chimeric EST sequences derived from assembly errors or ESTs containing non-conserved protein regions, as reported in other transcriptome analyses [34]–[36], it is also possible that some may constitute novel genes unique to this species.

Amongst ESTs derived from muscle tissue, coding sequences with homology to arginine kinase, ATP synthase, eukaryotic translation initiation factor, myosin heavy and light chain, sarcoplasmic calcium-binding protein, tropomyosin, and troponin were most abundant. Amongst ESTs derived from ovary tissue, coding sequences with homology to aldehyde dehydrogenase, ATP binding, cd63 antigen, cell division cycle, Chk1 checkpoint-like protein, e3 ubiquitin, eukaryotic translation initiation factor, ovary development-related protein, serine threonine-protein kinase, transmembrane protein, and WD repeat-containing protein were most abundant. Amongst ESTs derived from testis tissue, coding sequences with homology to eukaryotic translation initiation factor, kazal-type proteinase inhibitor, male reproductive-related protein, serine proteinase inhibitor, and viral A-type inclusion protein were most abundant. ESTs detected commonly across the 3 tissues included actins, elongation factors, eukaryotic translation initiation factor, heat shock protein, NADH dehydrogenase, reverse transcriptase, RNA-binding protein, senescence-associated protein, tubulin, ubiquitin and zinc finger protein (**Figure 3** **, Table S1**). Although this work was mainly focused on finding putative genes related with muscle development and growth, several putative functional transcripts identified here will lay the foundation for future studies aimed at investigating the role of sex determination, reproduction-related and xenobiotic genes which have been studied successfully in other species [26], [37], [38]. These findings could be the best source for deciphering the putative function of novel genes in each tissue but further studies need to be conducted to understand the molecular functions of specific reported genes.

### Gene Ontology assignments

**Figure 4:**
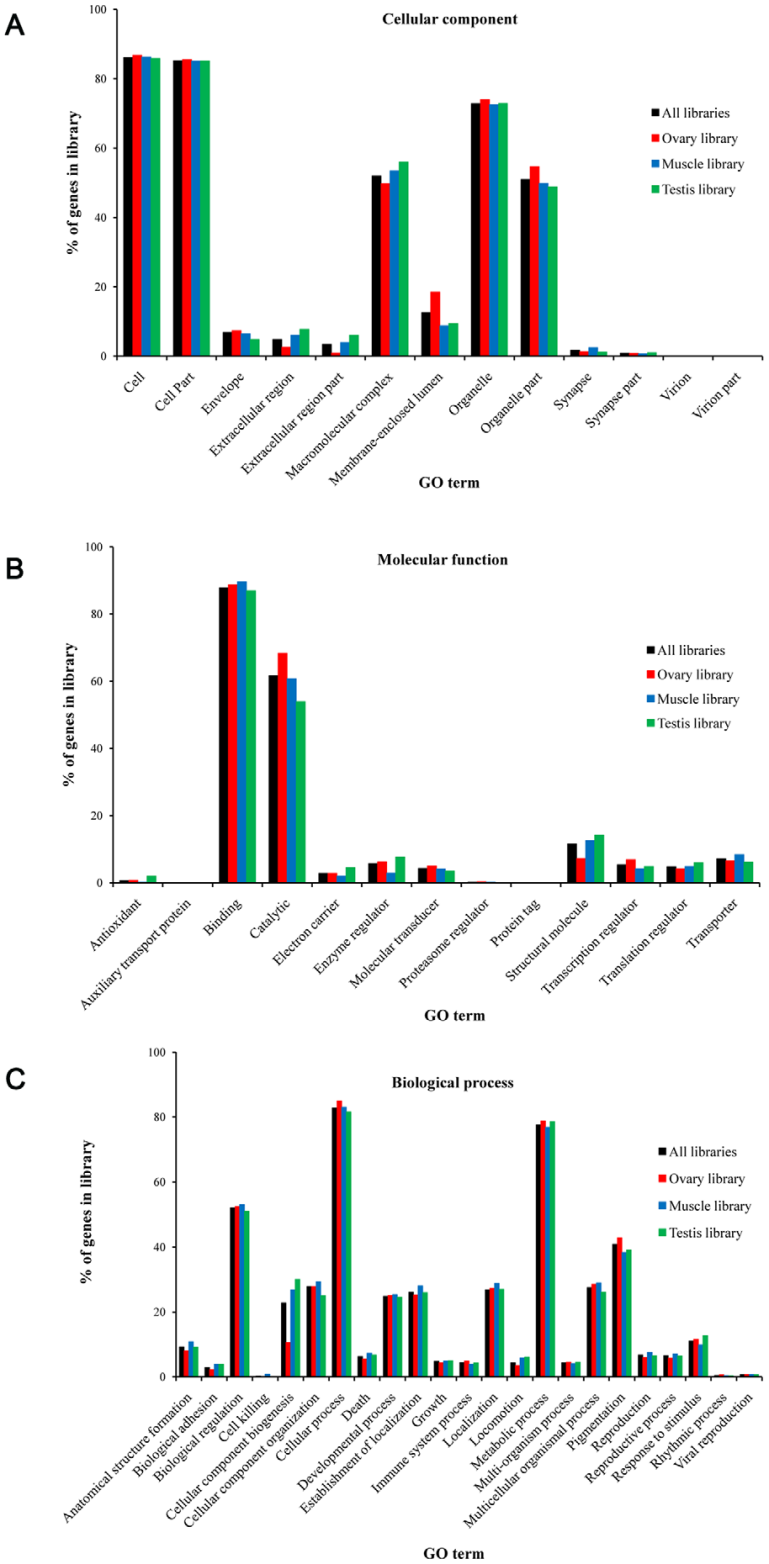
Gene ontology (GO) terms for the transcriptomic sequences of *M*. *rosenbergii* and comparison of among libraries. (A) cellular component, (B) molecular function and (C) biological process.

Gene Ontology (GO) terms could be assigned to 8411 *M*. *rosenbergii* contigs based on BLAST matches to proteins with known functions (**Figure 4** **, Table S2**). EST coding sequences were assigned to cellular components (4,550 sequences, **Figure 4A**), molecular function (6,055 sequences, **Figure 4B**) and biological processes (8,806 sequences, **Figure 4C**). Amongst ESTs assigned molecular functions, many were assigned binding (45.9%) or catalytic functions (32.3%), predominantly actin and zinc ion proteins (**Table S2**). Recent studies of crustaceans have highlighted the importance of actin in constructing muscle tissues and that it shows variable expression in different muscle types [39]–[42]. The cellular component assignments showed many EST coding sequences to likely possess cell (22.8%) and cell part (22.5%) functions, whilst those assigned biological functions were mostly predicted to be involved in cellular (17.6%) or metabolic processes (16.5%) including proteolysis, carbohydrate metabolism or oxidation-reductive functions. Analyses of the transcriptomes of other crustaceans have identified ESTs possessing similar arrays of potential metabolic functions [11], [14], [15], [43].

### KEGG analysis

Many of the coding sequences present in the *M*. *rosenbergii* EST contig dataset were identified to occur in KEGG pathways; metabolic pathways (*n* = 320), biosynthesis of secondary metabolites (*n* = 135), oxidative phosphorylation (*n* = 66), biosynthesis of phenylpropanoids (*n* = 59), and biosynthesis of alkaloids derived from histidine and purine (*n* = 51) (**Table S3**). Metabolic pathways, implicated in the kinetic impairment of muscle glutamine homeostasis in adult and old glucocorticoid-treated rats [44], showed the highest number of transcripts here. A skeletal muscle structure in rat intrauterine growth restriction indicated that changes in metabolic pathways were involved in obesity [45]. A total of 66 transcripts were involved in oxidative phosphorylation. The integrity of the inner membrane and the associated complexes is essential to oxidative phosphorylation to generate ATP to supply readily-available free energy for the body [46]. However, malfunction of oxidative phosphorylation could accentuate ATP depletion with the basic energy conservation system due to anoxic conditions in the tissues which could lead to metabolic failure [47].

Interestingly, we recovered a high number of transcripts that were mapped to the phenylpropanoids biosynthesis pathway (59). Phenylpropanoids not only play an important role in contributing to all aspects of plant responses towards biotic and abiotic stimuli [48] but also have a potential dietary importance from plant derived compounds [49]. A total of 51 transcripts also were predicted to the alkaloid biosynthesis pathway from histidine and purine in the *M*. *rosenbergii* EST contig dataset. Alkaloids, regarded as basic plant derived metabolites, are important components of plant defence, growth and development systems [50], [51]. In a study of sponges and ascidians, an abundance of alkaloids was reported that displayed biological activities such as metabolites [52]. Considering the omnivorous dietary habit of *M*. *rosenbergii*, finding these pathways was not surprising. Although not all of the major genes reported in putative KEGG pathways were found in the current study, this information provides insight into the specific responses and functions involved in molecular processes in *M*. *rosenbergii* metabolism and muscle contraction against biotic and abiotic stimuli.

### Protein domains

InterProScan searches identified 19,036 protein domains among the 8,411 *M*. *rosenbergii* contigs (**Table S4**). Consistent with similar analyses in insects and other crustaceans [14], [15], [28], domains that dominated occur in RNA-binding proteins, protein kinases and transcription factors (zinc finger domains) that are essential for cellular processing functions including signal transduction and transcription regulation, regulation of RNA stability and translation control (RNA recognition motifs), innate immunity, cell division, proliferation, apoptosis and cell differentiation [53], [54].

The most common DNA-binding motifs present in eukaryotic and prokaryotic transcription factors [55] were prevalent in the *M*. *rosenbergii* sequences, with 179 C2H2-type and 102 C2H2-like zinc finger (Znf) domains identified. Transcription factors usually contain several Znf domains capable of making multiple contacts with DNA [56], and can also bind to RNA and protein targets [57]. A total of 112 nucleotide-binding α-β plait domains found in RNA-binding domains from various ribonucleoproteins or in viral DNA-binding domains [58], [59] were predicted to exist among the *M*. *rosenbergii* EST coding sequences. In addition, 108 Armadillo-type fold and 84 Armadillo-like helical domains which form structural domains consisting of a multi-helical fold comprised of 2 curved layers of α-helices [60], were predicted (**Table 2**).

**Table 2:** Summary of top 20 domains predicted in *M*. *rosenbergii* sequences.

IPR accession	Domain name	Domain description	Occurrence
IPR000504	RRM_dom	RNA recognition motif domain	188
IPR017442	Se/Thr_prot_kinase-like_dom	Serine/threonine-protein kinase-like domain	180
IPR000719	Prot_kinase_cat_dom	Protein kinase, catalytic domain	180
IPR007087	Znf_C2H2	Zinc finger, C2H2-type	179
IPR004000	Actin-like	Actin-like	150
IPR002290	Ser/Thr_prot_kinase_dom	Serine/threonine-protein kinase domain	144
IPR012677	Nucleotide-bd_a/b_plait	Nucleotide-binding, alpha-beta plait	112
IPR016024	ARM-type_fold	Armadillo-type fold	108
IPR015943	WD40/YVTN_repeat-like_dom	WD40/YVTN repeat-like-containing domain	104
IPR011009	Kinase-like_dom	Protein kinase-like domain	104
IPR015880	Znf_C2H2-like	Zinc finger, C2H2-like	102
IPR017986	WD40_repeat_dom	WD40-repeat-containing domain	90
IPR011046	WD40_repeat-like_dom	WD40 repeat-like-containing domain	88
IPR002041	Ran_GTPase	Ran GTPase	86
IPR008271	Ser/Thr_prot_kinase_AS	Serine/threonine-protein kinase, active site	84
IPR013783	Ig-like_fold	Immunoglobulin-like fold	84
IPR011989	ARM-like	Armadillo-like helical	84
IPR023796	Sepin_dom	Serpin domain	79
IPR013083	Znf_RING/FYVE/PHD	Zinc finger, RING/FYVE/PHD-type	72
IPR016040	NAD(P)-bd_dom	NAD(P)-binding domain	72

Among *M*. *rosenbergii* EST coding sequences, 104 domains containing WD40/YVTN repeat-like sequences, 90 domains containing WD40-repeat sequences and 88 domains containing WD40 repeat-like sequences were predicted (**Table 2**). These domains are involved in a variety of functions ranging from signal transduction and transcription regulation to cell cycle control and apoptosis [61], [62]. A total of 86 Ran GTPase families which are involved in regulating GTP hydrolases [63], contain GTP-binding domains [64] and regulate receptor-mediated transport between the nucleus and the cytoplasm [65], [66] were also predicted, as were 84 immunoglobulin (Ig)-like fold domains (**Table 2**). Ig-like fold domains are involved in a variety of functions including cell-cell recognition, cell-surface receptors, muscle structure and the immune system [67], and are often involved with protein-protein interactions mediated by their β-sheets as in other Ig-like domains [67], [68]. Other domains identified abundantly included Serpin (*ser*ine *p*roteinase *in*hibitor) domains (*n* = 79) and NAD(P)-binding domains (*n* = 72) (**Table 2**). Interestingly, few PAZ (*n* = 3) or PIWI (*n* = 8) domains believed to be important components of the dsRNA-induced silencing complex were identified. The relative absence of ESTs with such domains is perplexing based on the detection of genes encoding Dicer and Argonaut type proteins in penaeid shrimp [69]–[71] and the clear demonstration of effective RNAi-mediated knockdown of gene expression in shrimp [70]. Similar transcriptome analyses of other tissues including haemocytes from the lymphoid organs for example that are primary mediators of pathogen defence responses [14], [15], [72] might be useful for indentifying if expression of ESTs encoding putative RNAi-related domains are more cell specific than domains required broadly for cell functioning. Although an original aim of this study was to identify candidate genes, gene families or gene domains potentially involved with growth phenotypes and/or other production traits important for *M*. *rosenbergii* aquaculture, none were differentiated from cell function or pathogen defence type activities. The identification of such ESTs has been confounded in most studies of shrimp to date focussing on the identification and characterisation of pathogen defence-related genes [14], [15], [72]. Thus genes mediating growth performance and potentially of value in selective breeding programs await discovery.

### Putative genes affecting muscle development and/or function

The *M*. *rosenbergii* EST sequence database was mined for coding sequences with domains involved potentially with muscle development and function (**Table 3**). Despite recent advances in sequencing technologies, few genes with such functions have been characterised from any crustaceans, and only 2365 ESTs assigned to *M*. *rosenbergii* and 5536 ESTs assigned to *Macrobrachium* were available in NCBI databases before this study. However, the 123,534 ESTs from the *M*. *rosenbergii* individuals selected from high and low growth performance cohorts should contain genes potentially expressed differentially and with functional characteristics suggestive of roles in muscle mass accumulation and other growth-related functions.

**Table 3:** Genes of interest for growth and muscle development in *M*. *rosenbergii* sequences.

Candidate genes	Contig IDs*	Length (bp)
Actin	A000585; A000586; A000587; A000588; A000763;A000764; A000765; A000766; A001338; A001339	1329; 1318; 1306; 1295; 1679; 1676; 1641; 1638; 930; 738
Alpha skeletal muscle	A000008; A000407; A000408; A000807; A002601; A002969	710; 1141; 1016; 1110; 1474; 1166
Calponin/calponin transgelin	A002718; A002875; A006133	1383; 1232; 518
Cyclophilin a	A001348; A001349	811; 850
Farnesoic acid O-methyltransferase	A002527	1587
Fatty acid binding protein	A004382	728
Lim domain binding	A000448	2610
Muscle lim protein	A000421; A000422; A000423; A000424; A000425	5788; 5694; 4595; 4501; 1716
Myosin heavy chain	A000009; A000016; A001103; A001282; A001283; A002073; A003870; A004348; A004442; A007715; A008193	612; 1510; 672; 942; 916; 711; 828; 733; 717; 383; 277
Myosin heavy nonmuscle or smooth muscle	A000018; A000968; A000969; A001363; A008338; A008352	1512; 5609; 2201; 730; 156; 148
Myosin light chain	A008264; A008271; A008339	220; 209; 155
Myosin light chain smooth muscle	A000639; A000783; A000785	3309; 2919; 1544
Profilin	A002454; A003703	1696; 872
Skeletal muscle actin 6	A000022; A000409; A005595; A006187; A007119; A008308; A008374	853; 522; 574; 512; 433; 177; 122
Transforming growth factor beta regulator 1	A006817	458
Tropomyosin	A000105; A000106; A000107; A000108; A000109; A000110; A000111; A000112; A000113; A000114; A000115; A000116; A001463; A002025; A002026; A007719	2777; 2769; 2770; 2768; 2762; 2760; 2773; 2775; 2765; 2767; 1962; 1954; 377; 1391; 110; 383

*Prefix “A” in ContigIDs indicates all merged contig from three libraries.

In the current study, both actin and myosin proteins including tropomyosin and troponin showed a high number of transcripts. It has been reported that actins are expressed in abundance as they are critical to formation of muscle filaments [73], [74]. Different actin isoforms have been identified in various crustaceans [40], and are likely to be involved in playing important roles in cytoskeletal structure, cell division and mobility, and muscle contraction [40]–[42]. The large super-family of myosin proteins interact with actin filaments by hydrolysing adenosine triphosphate that combine to form thick muscle filaments [75]. Myosin heavy chain (MHC) isoforms differ in their shortening velocity compared with other isoforms due to the enhanced ability of the myosin head to hydrolyse ATP [76]. Multiple MHC isoforms are expressed ubiquitously in all eukaryotic cells and they are the most abundant contractile protein present in skeletal muscle [77], [78]. If growth rates of *M*. *rosenbergii* are dictated primarily by the efficiency at which feed is converted into muscle mass, it is likely that myosin gene expression levels could provide a good molecular marker of individual growth potential, as found in the Atlantic pink shrimp *Farfantepenaeus paulensis* [79]. In studies of other crustaceans, high expression levels of genes encoding fast and slow myosin isoforms have been found to be accompanied by elevated expression of other genes encoding for example, actin, myofibrillar protein, tropomyosin, troponin I, and troponin T [80]–[82]. According to Perry et al. [83], differences in expression levels of myofibrillar protein isoforms correlate well with individual body size in crabs, with changes in expression spanning several orders of magnitude occurring at different life stages. Tropomyosins comprise a family of closely related proteins present both in muscle and non-muscle cells [84]. In striated muscle, tropomyosin mediates interactions between the troponin complex and actin to mediate muscle contraction [85]. A high number of actin and myosin protein transcripts observed here may regulate muscle development and function in *M*. *rosenbergii*. However, further studies are needed to confirm these observations.

High occurrence of calponin and transgelin was also observed in the transcriptome of *M*. *rosenbergii*. Calponin is a smooth muscle-specific protein capable of binding actin, tropomyosin and calmodulin and is also involved in mediating muscle contraction [86] as its interaction with actin inhibits actomyosin Mg-ATPase activity. In previous studies of invertebrates and vertebrates, caldesmon and calponin were shown to interact with actin, tropomyosin, and Ca^2+^-calmodulin [39], [41], [87]. In addition, transgelin is a calponin which is expressed exclusively in smooth muscle-containing tissues in adult animals and is one of the earliest markers of differentiated smooth muscle cells [88], [89].

The current study reports a number of putative genes, transcription factors, and early regulators that are potentially involved in muscle development and function in *M*. *rosenbergii*. Further studies need to be performed, however, to learn the molecular functions of these reported genes which were observed to be expressed more abundantly in adult female and male prawns compared with earlier developmental stages or slow growth performance individuals.

### Genes of interest related to growth

The transcriptome of *M. rosenbergii* was examined primarily to identify genes associated functionally with individual growth. For this reason, an EST dataset was compiled from tissues of individuals from high and low growth performance families (**Table 3**). Amongst these, a putative cyclophilin was identified. Although cyclophilins possess diverse functions and have been linked to innate immunity [90], [91] and testicular development [48], expression levels of cyclophilin-like proteins have also been found to be highly correlated with body-weight in the shrimp *P. monodon* [92].

Intracellular fatty acid-binding proteins (FABPs), identified in the current transcriptomic study, are members of a lipid-binding protein super-family that occur in both invertebrates and vertebrates, and together with acyl-CoA-binding protein (ACBP) are involved in lipid metabolism [93]. Few FABPs have been identified in invertebrates [93], [94], and their physiological roles remain largely unknown. However, in the locust *Schistocerca gregaria*, FABP expression has been reported to be strictly adult specific and is controlled by fatty acids in adult muscle [95]. Locust flight muscle employs fatty acids exclusively as the energy source for sustained flight and it is likely that FABP is involved in intracellular fatty acid transport [96].

In the current study, we found high occurrence of LIM domain proteins, which play important biological roles in cytoskeleton organisation, cell fate determination and organ development [97]. Previously, one LIM domain gene (ISL1) has been identified as a positional candidate for obesity and for controlling leptin levels, and is suggested to be involved in body weight regulation and glucose homeostasis [98]. In a study of the red crab *Gecarcoidea natalis*, two genes encoding LIM proteins, a paxillin-like transcript (pax) and a muscle LIM protein (mlp), were up-regulated in muscle of crabs in the wet season [99]. These proteins could play a fundamental role in muscle development and reconstruction, and their comparative up-regulation is consistent with a remodelling of leg muscle needed for migration during the wet season [99].

Physiologically, O-methyltransferase (OMT) plays an important regulatory role in plant and animal growth, development, reproduction and immune response [100], [101]. OMT transcripts observed in the current study could represent a potential candidate gene for developing novel traits in prawns. Methyl farnesoate (MF), the sesquiterpenoid precursor of insect juvenile hormone III (JH III), is produced and released by mandibular organs in decapod crustaceans [102]–[104]. The physiological function of MF, however, is not well understood in crustaceans, but by analogy with established functions of JH III in insects, MF has been suggested to play an important role in regulation of growth and reproduction in crustaceans [103], [105]. In some crustaceans, circulating titer and biosynthesis of MF appear to be correlated positively with maturation of the ovary [105], [106]. MF has also been suggested to play a role in delaying onset of molting in larval crustaceans [102], [106]. This evidence implicates MF in both crustacean growth and reproduction. Farnesoic acid O-methyltransferase (FAMeT; also known as S-adenosyl-methionine:farnesoic acid O-methyltransferase) is the enzyme that catalyses the final step in the MF biosynthetic pathway in crustaceans [107], [108]. Studies of crustacean FAMeT indicate that it may directly or indirectly (through MF) modulate reproduction and growth in crustaceans [109]–[112] by interacting with eyestalk neuropeptides as a consequence of its presence in neurosecretory cells in the X-organ-sinus gland. It is also believed that MF is the crustacean homolog for insect juvenile hormone, a molecule that may also regulate growth and reproduction in crustaceans [112]. If growth rates of *M*. *rosenbergii* are dictated primarily by the efficiency at which feed is converted into muscle mass, it is likely that FABP, LIM domain and FAMeT gene expression levels could provide candidate molecular markers of individual growth potential.

Another interesting finding in the current study is the expression of profilin, a small actin-binding protein found in eukaryotic cells that is critical for cytoskeletal dynamics [113], [114]. Profilins are potent regulators of actin filament dynamics and promote exchange of ADP to ATP on actin and by affinity to profilin–actin complexes for actin filament ends [115]. Profilins have diverse roles in cellular processes, including membrane trafficking, small-GTPase signalling and nuclear activities, neurological diseases, and tumor formation [116]–[118]. Genetic studies have shown the importance of profilins for cell proliferation and differentiation. Profilin gene disruption leads to grossly impaired growth, motility and cytokinesis, and embryonic lethality in multicellular organisms, for example in insects and mice [119]–[121].

The current study identified a number of putative genes that are potentially involved with growth in *M. rosenbergii*. However, further studies are needed to understand the molecular functions of these putative genes with growth performance and development in *M. rosenbergii*.

### Putative Molecular Markers

**Figure 5:**
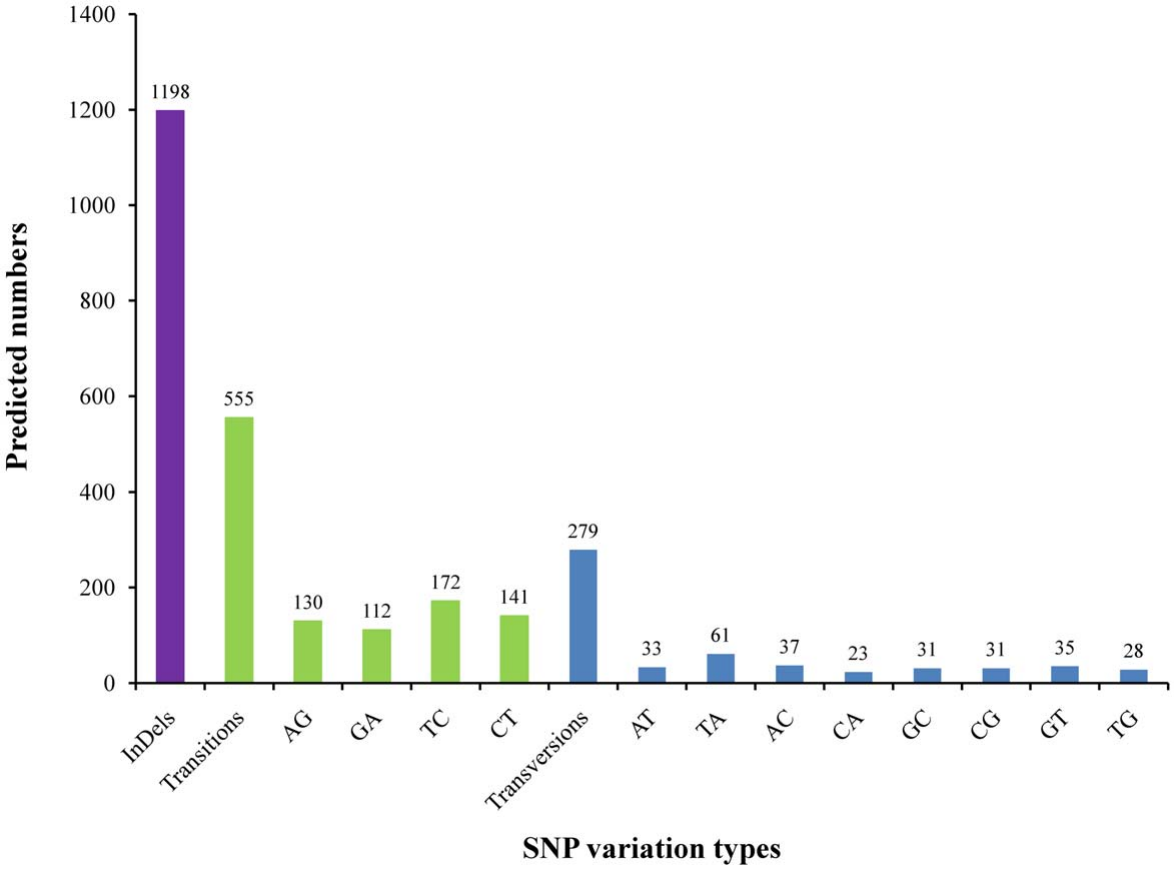
Distribution of putative single nucleotide polymorphisms (SNP) and indels in *M*. *rosenbergii* sequences.

**Figure 6:**
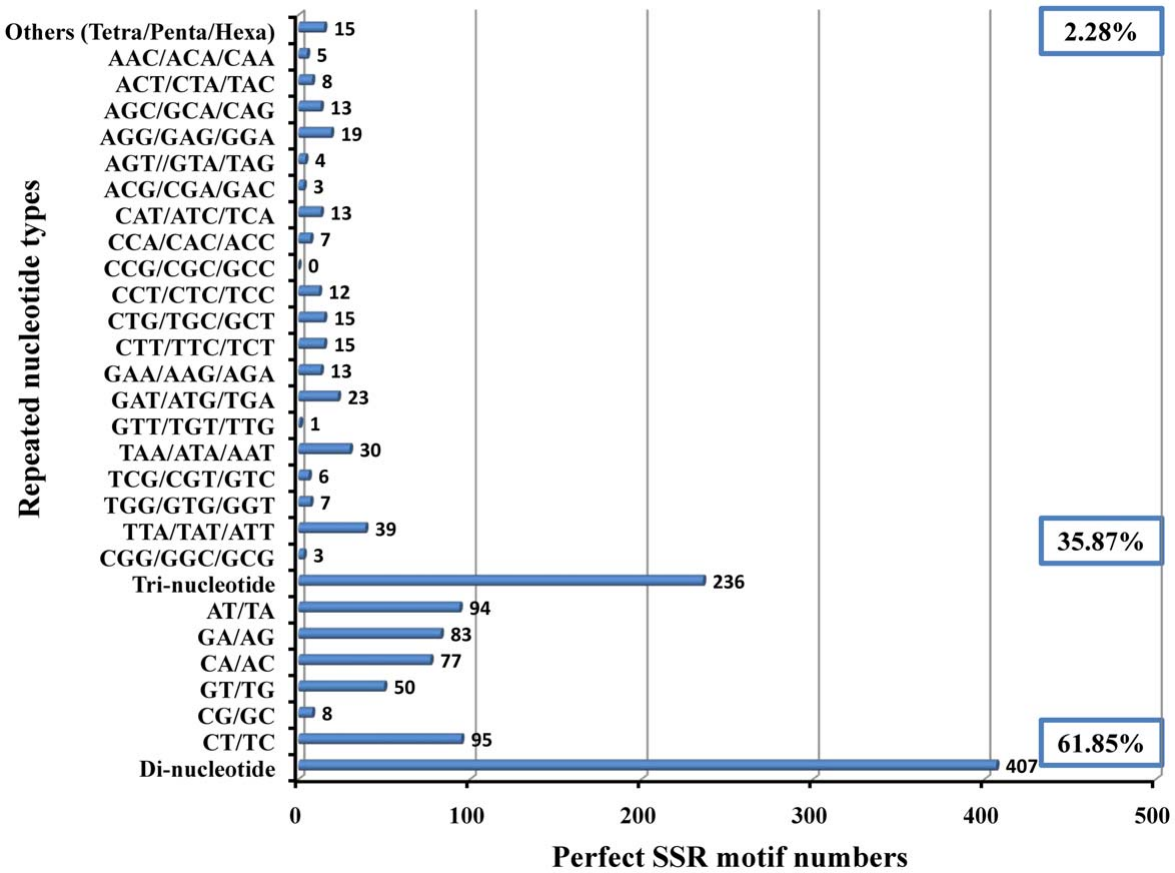
Distribution of simple sequence repeat (SSR) nucleotide classes among different nucleotide types found in *M*. *rosenbergii* sequences. Both contig and singleton sequences are used to predict the SSR loci.

SNPs in *M. rosenbergii* EST contigs were identified from alignments of multiple sequences used for contig assembly. Of the 834 SNPs detected, 555 were putative transitions (Ts) and 279 were putative transversions (Tv), giving a mean Ts : Tv ratio of 1.99 : 1.00 across the transcriptome (**Figure 5** **, Table S5**). The SNP types A ↔ G and C ↔ T were most common and SNP densities varied among genes, possibly due in part, to the effects of strong historical selection and the relative functional importance of individual genes. The Ts : Tv ratio can help identify genes affected by selection [122]. Although alignments also identified a total of 1198 indels across the transcriptome (**Figure 5** **, Table S5**), this must be treated with caution because of technical problems associated with 454 pyrosequencing [30], [37]. Moreover, a total 658 simple sequence repeats (SSRs) or microsatellites comprising 61.85% dinucleotide repeats, 35.87% trinucleotide repeats and 2.28% tetra/penta/hexa-nucleotide repeats were detected (**Figure 6** **, Table S6**) in the contigs as well as singletons.

PCR primers could be designed for almost all predicted polymorphic SSRs (**Table S6**) but these have yet to be validated as markers useful for examining *M*. *rosenbergii* adaptation and ecology as has been done with other non-model species [123]–[125]. In addition, SNPs and SSRs detected here are likely to be highly transferable to other closely related species as has been found for other crustacean species [125]–[127]. It is envisaged that the potential markers identified here within the ESTs will provide an invaluable resource for studying the evolution and molecular ecology of *Macrobrachium* species and for genome mapping and quantitative trait loci (QTL) analysis. However, many of the putative *M. rosenbergii* SNPs identified could simply represent allelic variants and future studies are planned to validate which are real. As ESTs were generated from 3 different tissue types, differential expression of different tissue-specific alleles is possible. However, this is rare as it requires somatic mutation or chimerisms between tissues.

### Conclusion

Here we report the first comprehensive EST dataset covering the transcriptome of the giant freshwater prawn *M*. *rosenbergii*, a non-model prawn species for which little molecular knowledge currently exists. The 123,534 putative ESTs (115,123 singletons and 8,411 contigs) identified and assembled will enable gene discovery in *M. rosenbergii*, assist in evolutionary studies and with the significant number of putative growth-related genes identified should facilitate genomics approaches to improving the growth performance of domesticated GFP stocks used for aquaculture. In addition, the large number of SNPs and SSRs detected provide targets for identifying polymorphisms across *M*. *rosenbergii* populations useful for parentage assignment and for managing inbreeding in cultured populations. Moreover, the EST sequences reported should prove invaluable for gene mining and annotation and phylogenetic analyses as well as provide a resource that can be exploited as molecular markers and in gene expression studies in this commercially important aquaculture species.

## Methods

### Tissue samples

*M*. *rosenbergii* with variable growth phenotypes were sampled from cohorts that were reared in a GFP stock improvement program in Vietnam [128]. Muscle and ovary tissue was sampled from adult females from high and low growth performance families and tissues preserved in 95% ethanol (454 sequencing run #1). Muscle was not sampled from males as their growth performance is confounded by social factors [6]. Muscle and ovary tissue from adult females and testis and eye-stalk tissue from adult males preserved in RNAlater (Ambion) were also analysed (454 sequencing run #2).

### RNA extraction

In 454 sequencing run #1, TRIzol® reagent (Invitrogen) [129] was used to extract total RNA from either muscle tissue or ovary tissue pooled from the three heaviest females from the high growth performance cohort and from the three lightest females from the low growth performance cohort. In 454 sequencing run #2, total RNA was extracted similarly from muscle/ovary (female) and testis/eye-stalk (male) from groups of three prawns in the same growth categories as used in 454 sequencing run #1. Total RNA was purified further using a RNA Easy Kit (QIAGEN). RNA yields and quality were checked using both a Bioanalyzer nanochip (Agilent) and a Nanodrop spectrophotometer (Thermo). Equal amounts of total RNA purified from each tissue type were pooled and mRNA was isolated using the MicroPoly(A) Purist™ Kit (Ambion) according to the manufacturer's protocol.

### Library construction and 454 pyrosequencing

mRNA purified from pooled muscle, ovary, testis and eye-stalk total RNA from males and females of high and low growth performance were sent to the Australian Genome Research Facility (AGRF), Brisbane, Australia, for cDNA synthesis using a cDNA Rapid Library Preparation Kit (Roche) and subjected to 454 GS-FLX sequence analysis. Due to issues with poor RNA and cDNA quality and low yields from eyestalk tissue, this tissue was excluded from the cDNA library. The cDNA library sequenced thus comprised a pool of cDNAs prepared from muscle tissue from the three heaviest females, ovary tissue from the three heaviest and three lightest females and testis tissue from the three heaviest males. Each cDNA was normalized prior to pooling to reduce sequence coverage of high copy number mRNAs and samples tagged for downstream identification. cDNA yields were quantified using a Quant-iT RiboGreen fluorometer (Invitrogen) and average lengths were determined by analysis of an aliquot (1 µl) on the Bioanalyzer (Agilent) using a LapChip 7500. Oligonucleotide adapters A and B (Roche) were ligated to cDNA 5′ and 3′ ends and cDNA was amplified by PCR using the same primers and a proof reading polymerase. Emulsion PCR (emPCR) set up, breaking, enrichment and pico-titer plate (PTP) loading steps were performed according to Roche protocols [31]. Each of the two sequencing runs employed half of a PTP and was sequenced twice using Roche 454 GS FLX chemistry (Roche) according to the manufacturer's protocol.

### Sequence cleaning and assembly

All sequence reads taken directly from the 454 GS-FLX sequencer were run through the sff file program (Roche) to remove sequencing adapters A and B, poor sequence data and barcodes. Contigs and singletons were renamed in a format ‘A (M, O, T)_000001’ where prefix ‘A’ was used for all assembled contigs derived from M, O, T cDNA libraries, with M (Muscle), O (Ovary), and T (Testis) standing for an individual library and assembly, and 000001 standing for the first arbitrary contig assignment number. In the case of singletons, the same prefix codes (A, M, O, T) for cDNA library origin(s) were added in front of each read name (e.g. A_G1OH9PT01AF0I7). Sequences containing homopolymers of a single nucleotide comprising >60% of the read and that were >100 nucleotides in length were discarded. Trimmed sequences were assembled *de novo* using the default parameters of Newbler 2.5.3 (Roche). Each dataset of mRNA sequences from muscle, ovary and testis tissue was considered separately as being representative of the transcriptome of that tissue type at the time of sampling. On the assumption that some transcripts would be replicated across tissue-type datasets, these were merged in the combined dataset. After initial quality filtering, AGRF provided assembled contig and singleton datasets for analysis. All *M*. *rosenbergii* EST sequences obtained were submitted to NCBI Sequence Read Archive under Accession no. SRP007672.

### Annotation of mRNAs

BLASTx searches [130] of the GenBank non-redundant (nr) database hosted by the National Center for Biotechnology Information (NCBI) (http://www.ncbi.nlm.nih.gov/) were performed on all contigs and singletons to identify putative mRNA functions (E-value threshold <1e^–5^) as well as new ESTs. Numbers of ESTs that were either unique or shared among the libraries were visualized using a 3-way Venn diagram constructed using Venny [131]. Total EST numbers in the Venn diagram quadrants excluding abundant ESTs for ribosomal proteins counted redundant ESTs only once. The Blast2GO software suite [132], [133] was used to predict functions of individual ESTs, assign Gene Ontology terms [134], [135], and to predict metabolic pathways using Kyoto Encyclopaedia of Genes and Genome (KEGG) [136], [137]. To identify protein domains, all translated sequences were interrogated against the InterPro databases (http://www.ebi.ac.uk/Tools/pfa/iprscan/) using the InterProScan tool [138]. The numbers of contigs annotated with each GO term for each library were quantified using WEGO [139].

### Identification of EST-SSR motifs and EST-SNPs

All EST sequences were searched for SSR motifs using the QDD program [140]. Default settings were employed to detect perfect di-, tri-, tetra-, penta-, and hexa-nucleotide motifs (including compound motifs). To be assigned, dinucleotide SSRs required a minimum of 6 repeats, and all other SSR types a minimum of 5 repeats. The maximum interruption between 2 neighbouring SSRs to consider it being a compound SSR was set at 100 nucleotides. Perl script modules linked to the primer modelling software Primer3 [141] were used to design PCR primers flanking for each unique SSR region identified.

Multiple nucleotide sequence alignments of contigs identified among the EST libraries derived from individual *M. rosenbergii* with divergent growth phenotypes were undertaken to identify putative SNPs. Alignments employed methods developed previously for plants and other species of agricultural importance [33], [127], [142] and included assessments of raw data alignments used in the initial assembly of contigs. Since no reference sequences were available, SNPs were identified as superimposed nucleotide peaks where 2 or more reads contained polymorphisms at the variant allele. SNPs were identified using default parameters in gsMapper (Roche) to align contigs from the individual and merged tissue type and prawn phenotype datasets and SNPs were predicted with high confidence when (i) the difference existed in at least three non-duplicated reads, (ii) the difference occurred in both the forward and reverse sequence reads unless present in at least seven same direction reads with quality scores over 20 (or 30 if the difference involves a 5-mer or more) and (iii) the difference comprised a single-base overcall or undercall forming a consensus differing from the each contig reference. Indels were segregated into simple types containing an insertion or deletion of at least one nucleotide compared with the reference sequence or complex types also containing nucleotides substitutions.

For the merged EST dataset, loose or strict criteria to maximize the discovery of rare alleles or to minimize the possibility of false-positive identifications were not considered [26], [127]. In addition, only an overall transition vs transversion (Ts/Tv) ratio was calculated across the dataset.

## Supporting Information

Table S1 **Summary of BLASTx results for contigs and singletons of** ***M*** **.** ***rosenbergii*** **.**
(XLSX)

Table S2 **Gene Ontology of** ***M*** **.** ***rosenbergii*** **contig sequences.**
(XLSX)

Table S3 **KEGG summary of** ***M*** **.** ***rosenbergii*** **contig sequences.**
(XLSX)

Table S4 **InterProScan domain search of** ***M*** **.** ***rosenbergii*** **contig sequences.**
(XLSX)

Table S5 **Putative SNPs and Indels in** ***M*** **.** ***rosenbergii*** **contig sequences.**
(XLSX)

Table S6 **Putative microsatellite loci in** ***M*** **.** ***rosenbergii*** **contig and singleton sequences.**
(XLSX)
